# Complete chloroplast genome of medicinally important poisonous shrub *Adenium obesum* (Forssk.) Roem. & Schult. (Apocynaceae)

**DOI:** 10.1080/23802359.2019.1710292

**Published:** 2020-01-14

**Authors:** M. Ajmal Ali

**Affiliations:** Department of Botany and Microbiology, College of Science, King Saud University, Riyadh, Saudi Arabia

**Keywords:** Chloroplast genome, medicinal plant, *Adenium obesum*, Apocynaceae

## Abstract

The complete chloroplast genome of medicinally important plant *Adenium obseum* (Forssk.) Roem. & Schult. (Apocynaceae) was sequenced. A total of 5,028,192,488 paired-end reads were obtained. One-hundred-and-twenty-seven unique coding genes including 96 protein-coding, 28 tRNA, and 3 rRNA genes were annotated. The length and GC content of the plastome were 154,437 bp (GenBank accession number MN765097) and 38.1%, respectively.

The drought-deciduous succulent poisonous shrub *Adenium obesum* (Forssk.) Roem. & Schult. (commonly known as Desert Rose) belongs to the family Apocynaceae, subfam. Apocynoideae, tribe Nerieae, subtribe Neriinae. In the wild, it is mainly distributed in the tropical and subtropical regions of Africa to Arabia and used in traditional medicines and contains toxic cardiac glycosides (Versiani et al. 2014). The recent advances in next-generation sequencing (NGS) such as Illumina, PacBio, and Nanopore sequencing platforms, and bioinformatics tools and techniques for the sequence data analysis in the current decade (Shendure et al. 2017) have greatly helped to understand species relationships in the tree of life to gene function and genomes evolution (Daniell et al. 2016; Leebens-Mack et al. 2019), and have transformed the discipline from phylogenetics into phylogenomics (McKain et al. 2018). The genus *Adenium* is very closely related to *Nerium* (subfam. Apocynoideae, tribe Nerieae, subtribe Alafiinae), the morphological characters such as inflorescence, corolla, carpels, fruits and the usually slightly exserted appendices of the stamens of *Adenium* and *Nerium* resembles each other (Endress et al. 2014). Hence, the complete plastome of *A. obesum* was sequenced for the first time in order to assess the systematic relationships.

The whole chloroplast genome was sequenced in the present study on a Illumina sequencing platform from the DNA extracted from the young and green leaves of *A. obesum* collected from the botanical garden of the King Saud University [Voucher: MAA 15 (KSUH), (24°43′10′′N, 46°36′55′′E), Riyadh, Saudi Arabia using DNeasy DNA extraction kit (QIAGEN, Hilden, Germany). The total number of bases, reads, GC (%), Q20 (%), and Q30 (%) were calculated. The filtered data statistics revealed 3,785,889,890 total read bases with 25,235,946 total reads with Q30 (the percentage of bases that have a quality score of 30 or above) being 95.96%. *De novo* assembly was performed using NOVOPlasty (Dierckxsens et al. 2017). A total of 127 unique coding genes including 96 protein-coding, 28 tRNA, and 3 rRNA genes were annotated using AGORA (Jung et al. 2018). The length and GC content of the plastome were 154,437 bp (GenBank accession number MN765097) and 38.1%, respectively.

A number of studies based on both morphological as well as DNA sequence data have previously been undertaken to resolve the phylogeny of the family Apocynaceae, the recent classification contains a total of 366 genera, which are recognized and placed within five subfamilies, 25 tribes, and 49 subtribes (Endress et al. 2014), including plastome phylogeny of Apocynaceae (McKain et al. 2018), revealing relationship among the tribe and subtribe. In the present analysis, the BLAST search of the chloroplast genome sequence of *A. obesum* was performed. Followed by the phylogenetic tree reconstruction with the available representative chloroplast genome sequences [*Secamone afzelii* (Roem. & Schult.) K. Schum. (KF539845.2, subfam. Secamondoideae), *Asclepias syriaca* L. (NC_022432 subfam. Asclepiadoideae, tribe Asclepiadeae, subtribe Asclepiadinae), *Amphineurion marginatum* (Roxb.) D. J. Middleton (MG963253 (subfam. Apocynoideae, tribe Apocyneae, subtribe Amphineruiinae), *Periploca sepium* Bunge (KJ953910 subfam. Periplocoideae) *Alafia barteri* Oliv. (MG963238, subfam. Apocynoideae, tribe Nerieae, subtribe Alafiinae), *Nerium oleander* L. (KJ953907, subfam. Apocynoideae, tribe Nerieae, subtribe Alafiinae), *Adenium obesum* (Forssk.) Roem. & Schult. MN765097, subfam. Apocynoideae, tribe Nerieae, subtribe Neriinae), *Alstonia scholaris* (L.) R. Br., MG963247 (subfam. Rauvolioideae), *Gelsemium sempervirens* (L.) J. St.-Hil. (MG963263, family Gelsemiaceae, outgroup)] using NGPhylogeny.fr (Lemoine et al. 2019) to unravel (a) its phylogenetic relationship within the family and (b) evolution of chloroplast genome in the family Apocynaceae, which resulted in the phylogenetic relationships of the family to be consistent with previous reports (McKain et al. 2018) and its nesting with subfam. Apocynoideae, tribe Nerieae (Figure 1) as per the previous treatment based on morphological and molecular data (Endress et al. 2014).

**Figure 1.**
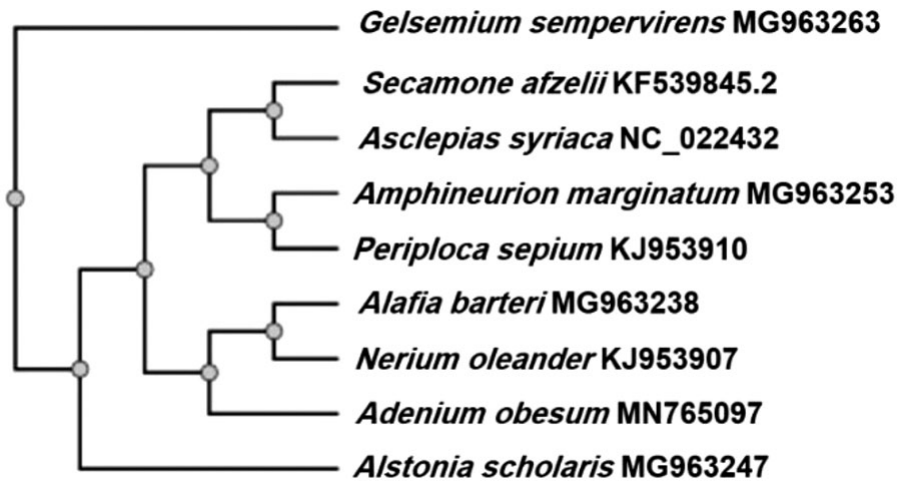
The phylogenetic analysis of chloroplast genome of *A. obesum* with the representative of the subfam. Secamondoideae, Asclepiadoideae, Apocynoideae, Periplocoideae, Rauvolioideae (Family Apocynaceae) outgropup at *G. sempervirens* (Family Gelsemiaceae). Number next to taxon indicates GenBank accession number.
